# Congenital Short QT Syndrome

**Published:** 2010-02-01

**Authors:** Lia Crotti, Erika Taravelli, Giulia Girardengo, Peter J Schwartz

**Affiliations:** 1 Section of Cardiology, Department of Lung, Blood and Heart, University of Pavia, Pavia, Italy; 2 Department of Cardiology, IRCCS Fondazione Policlinico S. Matteo, Pavia, Italy; 3 Molecular Cardiology Laboratory, IRCCS Fondazione Policlinico S. Matteo, Pavia, Italy; 4 Laboratory of Cardiovascular Genetics, IRCCS Istituto Auxologico, Milan, Italy; 5 Cardiovascular Genetics Laboratory, Hatter Institute for Cardiovascular Research, University of Cape Town, Cape Town, South Africa; 6 Sudden Death Chair, King Saud University, Riyadh, Saudi Arabia

**Keywords:** Short QT Syndrome, sudden cardiac death, channelopathies, ICD

## Abstract

The Short QT Syndrome is a recently described new genetic disorder, characterized by abnormally short QT interval, paroxysmal atrial fibrillation and life threatening ventricular arrhythmias. This autosomal dominant syndrome can afflict infants, children, or young adults; often a remarkable family background of cardiac sudden death is elucidated. At electrophysiological study, short atrial and ventricular refractory periods are found, with atrial fibrillation and polymorphic ventricular tachycardia easily induced by programmed electrical stimulation. Gain of function mutations in three genes encoding K^+^ channels have been identified, explaining the abbreviated repolarization seen in this condition: KCNH2 for I_kr_ (SQT1), KCNQ1 for I_ks_ (SQT2) and KCNJ2 for I_k1_ (SQT3). The currently suggested therapeutic strategy is an ICD implantation, although many concerns exist for asymptomatic patients, especially in pediatric age. Pharmacological treatment is still under evaluation; quinidine has shown to prolong QT and reduce the inducibility of ventricular arrhythmias, but awaits additional confirmatory clinical data.

##  Introduction

The relation between the QT interval and sudden cardiac death has been known for 50 years, but only recently it has been demonstrated that not only a long QT interval but even a short QT interval is related to an increased risk of sudden cardiac death [1].

The Short QT Syndrome (SQTS) is a new clinical entity first described by Gussak et al [2] in 2000 as a syndrome related to sudden cardiac death. SQTS typically occurs in young individuals with structurally normal hearts and with increased risk of arrhythmias and sudden death. The clinical features of this newly identified syndrome overlap those of other genetic arrhythmogenic diseases linked to ion channel abnormalities, such as the Long QT Syndrome, the Brugada syndrome and Catecholaminergic Polymorphic Ventricular Tachycardia. Three distinct genes, all encoding ion channels, have been identified in association with SQTS, that can therefore be defined as a genetically heterogeneous syndrome.

This review will address the genetic basis, the clinical features and the diagnosis of this disease, with specific attention to the therapeutic options.

## History

The relation between a Short QT interval and sudden cardiac death was originally suspected by Algra et al [3] in a retrospective study in which the QT interval of 6693 subjects was measured in Holter recordings. It was found that susceptibility to arrhythmias and sudden cardiac death was associated not only with long QT intervals, but also with short QT intervals.

The SQTS was originally described by Gussak et al [2] in two cases: a 17 year-old girl who went into spontaneous atrial fibrillation with rapid ventricular response following a laparoscopic cholecystectomy and a 37-year-old female who died suddenly after two syncopal events. In both cases the ECG showed a markedly short QT interval (QTc 280 ms), suggesting that short QT may be responsible for electrical instability and susceptibility to develop atrial and/or ventricular arrhythmias. However, only with the study by Gaita et al [4] published in 2003, the short QT syndrome was considered as a new clinical entity with an autosomal dominant inheritance. Seven affected members of two distinct European families were described and a QT interval less than 280 ms was associated with syncope, palpitations and sudden cardiac death [4]. Four patients underwent electrophysiological evaluation including programmed ventricular stimulation and short atrial and ventricular refractory period, linked to ventricular and atrial vulnerability to fibrillation, were documented [4].

## Genetics

Three main genetic variants have been described in the short QT syndrome, involving potassium channel genes also associated with the long QT syndrome (LQTS). However, while mutations on the potassium channel genes causing LQTS are loss of function mutations, those observed in the short QT syndrome are gain of function mutations causing a shortening of the action potential duration [1].

SQT1 is caused by mutations on KCNH2 (HERG), the gene also responsible for LQTS, type 2. The genetic screening in the first two reported families with Short QT Syndrome and sudden cardiac death, led to the identification of two different missense mutations on KCNH2 that resulted in the same amino acid change of the cardiac I_kr_ channel [5]. In one family, a missense mutation with a cytosine to guanine substitution at nucleotide 1764 on KCNH2 was reported, while in the second family a cytosine to adenine substitution at the same nucleotide was observed. Both mutations induced the substitution of the asparagine at codon 588 with a positive charged lysine (KCNH2-N588K).

**Figure 1:**
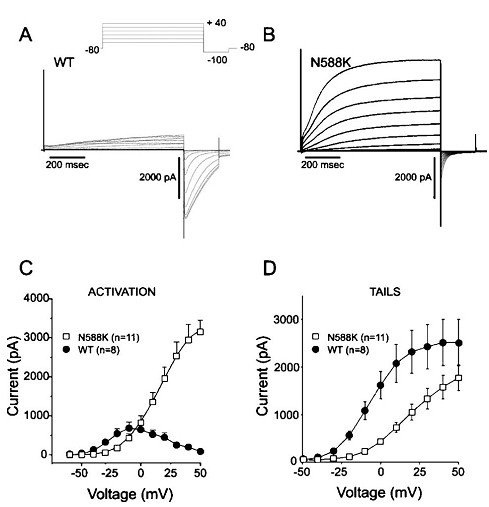
Mutation N588K removed rapid inactivation of the current in a physiological range of membrane potentials. (A) Wild-type KCNH2/KCNE2 currents elicited by 800-ms depolarizing pulses in TSA201 transfected cells. Activation currents were elicited by depolarizing pulses in +10 mV increments. A large inward tail current is observed upon repolarization to -100 mV. (B) Same protocol as in A applied to N588K. Tail currents were reduced by the mutation. (C) Current-voltage relationship for steady state current measured at the end of the activating pulse. Current amplitude was largest in N588K despite transfection with equivalent molar ratios. (D) Tail current-voltage relationship as a function of the activating step potential. WT: n =8, N588K: n =11. Reproduced with permission from: Cordeiro JM, Brugada R, Wu YS et al.  Modulation of Ikr inactivation by mutation N588K in KCNH2: a link to arrhythmogenesis in short QT syndrome. Cardiovascular Research 2005: 67: 498-509. Oxford University Press / European Society of Cardiology.

To further elucidate the mechanism of QT interval shortening, the mutated KCNH2 channel (N588K) was co-expressed with and without the beta-subunit MiRP1 (KCNE2) in human embryonic kidney cells (TSA201) and patch-clamp experiments were performed [6]. Whole-cell recordings demonstrated that the mutation causes a loss of the normal rectification of the current at plateau voltages, which results in a significant increase of IKr during the action potential plateau and leads to an abbreviation of the action potential and refractoriness [6] (Figure 1). Additionally, the N588K currents showed a much larger relative current at the initial phase of the action potential [5]. Shortening of ventricular AP is supposed to be linked to a shortening of the effective refractory period, thus causing an increased ventricular and atrial susceptibility to premature stimulation, leading to AF and VF.

Genetic heterogeneity in the SQTS was soon made evident by the findings of Bellocq et al, who identified a mutation on KCNQ1 (V307L) in a 70-year-old patient with a QTc 302 ms and aborted sudden cardiac death [7]. The mutation, expressed in COS-7 cells, caused a gain of function of I_ks_, resulting in an abbreviation of the action potential duration and shortening of the QT interval  [7]. The KCNQ1 gene is therefore not only responsible for LQTS, type 1, but also for SQTS, type 2 (SQT2). A second mutation (V141M) in the S1 segment of KCNQ1 was identified the following year by Hong et al [8] in a baby girl born at 38 weeks after induction of delivery that was prompted by bradycardia and irregular rhythm. The ECG revealed atrial fibrillation with slow ventricular response and short QT interval. To characterize the physiological consequences of the V141M mutation, Xenopus oocytes were injected with cRNA encoding wild-type KCNQ1 or mutant V141M KCNQ1 subunits, with or without KCNE1. Computer modelling showed that the mutation would shorten action potential duration of human ventricular myocytes and abolish pacemaker activity of the sinoatrial node  [8].

SQTS, type 3 (SQT3) was described by Priori et al [9] in 2005 and was associated with a gain of function mutation in the KCNJ2 gene, encoding for the strong inwardly rectifying channel protein Kir2.1, also involved in the Andersen-Twail Syndrome. The two affected members of a single family had a G514A substitution in the KCNJ2 gene that resulted in a change from aspartic acid to asparagine at position 172 (D172N) [9]. Functional characterization of the mutation demonstrated a significant increase in the outward I_k1_ current [9].

Recently, Antzelevitch et al reported three cases in which the Brugada Syndrome phenotype and a family history for sudden cardiac death was associated with QTc ≤ 360 ms [10]. In these three cases a mutation in genes encoding the α1- or β2b- subunits of the cardiac L-type calcium channel were identified and specifically  a mutation on CACNB2b (S481L) and two mutations on CACNA1C (A39V and G490R) [10]. To determine the contribution of each mutation to the clinical phenotype, each of the WT and mutated CACNA1C and CACNB2b mutations were expressed in CHO cells. The results of patch-clamp experiments indicate that all the mutations cause a major loss of function in calcium channel activity [10]. The QTc observed in these three cases and in affected family members ranged from 330 to 370 ms, a QTc longer than what was observed in other SQTS families. Accordingly, it seems premature to consider CACNB2b and CACNA1C as SQTS genes, but it is probably more appropriate to define what was observed by Antzelevitch et al as a new clinical entity, characterized by overlapping phenotypes.

In summary, three main potassium channel genes have been associated to Short QT Syndrome. Genetic analysis may help identifying silent carriers of SQTS-related mutations; however, the risk of cardiac events in genetically affected individuals with a normal ECG is currently not known. Similarly, given the limited number of patients with SQTS so far identified, at present, genetic analysis does not contribute to risk stratification.

## Clinical findings

Short QT Syndrome has been described in very few families world-wide; therefore, all the information available is based on less than thirty cases. All patients described presented with a QTc below 320 msec and structural heart disease was excluded. Patients with this disease are likely to be at high risk for syncope and/or sudden cardiac death due to ventricular tachyarrhythmias and episodes of atrial fibrillation are frequently documented at different ages even in adolescents and children. No information is available on whether specific triggers may precipitate cardiac events, as cardiac arrest has occurred both at rest and under stress.

The largest study published so far on SQTS [11], included 29 patients, 25 belonging to eight SQTS  families and 4 sporadic cases, all with family and/or personal history of cardiac arrest (CA)/ sudden cardiac death (SCD) and documented short QT interval on the surface ECG. The first clinical presentation was sudden cardiac death in one third of cases. AF was also observed quite frequently in young patients and presumably linked to the presence of short refractory periods in the atria. Syncope was observed as a first clinical presentation less frequently (14%) and the age of presentation was quite variable from 4 months to 62 years.

In three cases the first clinical manifestation occurred in the first year of life and two of them had aborted sudden death, suggesting a possible role for SQTS in some cases of sudden infant death syndrome (SIDS), the most frequent cause of mortality in the first year of life. Actually, the largest study to date, conducted on a cohort of SIDS victims [12] identified a disease-causing mautation in LQTS genes in about 10% of cases, and one of those mutations, the KCNQ1-I274V causes a gain of function in IKs predicting a short QT phenotype [13]. Therefore, SQTS can be considered, together with other channelophaties, as a contributing cause of SIDS.

In summary, the Short QT Syndrome - initially described as a very severe disease - shows a wide range of clinical manifestations varying from atrial to ventricular fibrillation and sudden death.  Additionally, also the age of occurrence of the first cardiac event can be very different, ranging from the intrauterine period to 70 years old.

## Diagnosis

Diagnostic criteria for the Short QT Syndrome are not available and even the lower limit of the normal QT interval has not been fully established. In the reported cases of Short QT Syndrome, the heart rate corrected QT (QTc) interval using the Bazett formula was always below 320 ms, except for the three cases, reported by Antzelevitch et al in which the Brugada Syndrome phenotype and a family history for sudden cardiac death were associated with QTc ≤ 360 ms [10].

To better define the distribution and prognostic significance of short QT intervals, two different approaches have been used. Initially, a population of patients with idiopathic ventricular fibrillation (IVF) was studied to determine if they had shorter QT intervals compared to healthy controls [14]. Subsequently, two "normal populations" with known QTc values were followed prospectively to identify a possible correlation between short QTc and risk of sudden cardiac death [15,16].

In the first study, the ECGs of 28 consecutive patients with IVF were compared to those of 280 age- and gender-matched healthy controls [14]. Interestingly, QTc values below 360 ms were found more frequently among males with IVF than controls, but this was not true for females. Additionally, "short" QTc values were not rare among healthy controls, especially at slow heart rates, therefore further studies were needed to establish when a given QT was really "too short".

The first "normal population" studied, includes 12012 subjects who underwent routine medical examinations for occupational reasons [15]; the shortest QTc encountered in this cohort was 335 ms and the lowest 0.5 centile was 360 ms. Information about subsequent survival was available for 36 of the 60 subjects with a QTc ≤360 ms and none of these died during the 7.9±4.5 years subsequent follow-up [15]. The conclusion of this study was that a QTc≤330 ms is extremely rare in healthy subjects, and the presence of a QT interval in the lowest 0.5 centile of normal ranges does not imply a significant risk of sudden death [15].

More recently, a middle-aged Finnish population (n=10822) was studied and followed up for 29±10 years [16]. The prevalence of QTc<320 ms was 0.1%, using the Bazett formula; the prevalence of QTc<340 ms was 0.4%. All-cause or cardiovascular mortality did not differ between subjects with very short (<320 ms) or short (<340 ms) QT interval and those with normal QTc (360-450 ms) [16]. There were no sudden cardiac death, cardiac arrests or documented ventricular tachyarrhythmias among subjects with QTc <340 ms; while subjects with a QTc >450 msec had greater all-cause and cardiovascular mortality than those with normal or short QT intervals [16].

These studies suggest that the presence of a short QT in the ECG is not sufficient to make a diagnosis of Short QT Syndrome.

**Figure 2:**
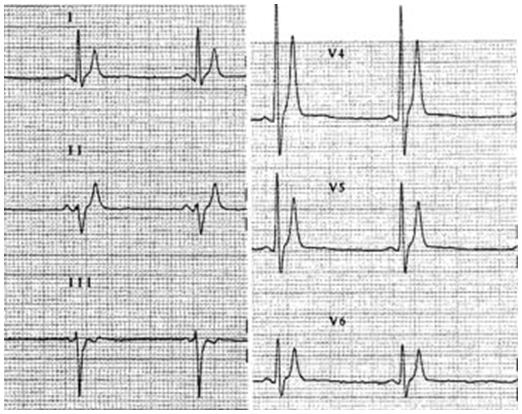
Twelve lead ECG showing typical SQT1 features: tall, narrow and peaked T waves, QT 280 ms. Reproduced with permission from:  Gaita F, Giustetto C, Bianchi F et al. Short QT Syndrome: a familial cause of sudden death. Circulation. 2003: 108: 965-70.

Apart from constantly short QTc intervals, affected patients have in common a short or even absent ST segment, with the T wave initiating immediately from the S wave. In SQT1 patients (those with a mutation on KCNH2) the T waves in the precordial leads, appear often tall, narrow and symmetrical, with a relatively prolonged Tpeak-Tend interval [4] (Figure 2). This may indicate an augmented transmural dispersion of repolarization [17,18]. In the two SQT2 patients described (mutation on KCNQ1) the T waves appear to be symmetrical, but not as tall and narrow [7,8]. By contrast, the two related patients with a mutation on KCNJ2 gene (SQT3), showed an asymmetrical pattern with a less steep ascending section of the T wave, which is followed by a rapid descending and terminal phase of the T wave [9]. This pattern could be justified by the electrophysiological consequences of the mutation identified, as simulations of the cardiac action potential propagation showed a different repolarization of the KCNJ2 mutant with a sudden acceleration of the final phase of the action potential repolarization [9]. Finally, a further relevant feature in patients with SQTS is the lack of adaptation of the QT interval to heart rate [19,20].

Eleven SQTS patients all carrying the KCNH2-N588K mutation, underwent an invasive electrophysiological analysis. During programmed atrial and ventricular stimulation, the atrial and ventricular effective refractory periods were extremely short (atrial refractory period 141±18 ms and ventricular effective refractory periods  147±18 ms) [21]. Additionally, in a very high percentage of the patients, ventricular tachyarrhythmias, predominantly ventricular fibrillation/ventricular flutter, were inducible (10/11 patients 91%) [21]. Belloq's SQT2 patient had effective refractory periods =180 ms and no inducible VF with double premature stimuli at coupling intervals ≥180 ms [7]. Priori's SQT3 patient had an effective refractory period of 160 ms and VF inducible with three premature stimuli [9]. Thus, SQTS patients with a variety of ion channel defects display the common finding of short atrial and ventricular effective refractory periods and, in some cases, inducible atrial and ventricular tachyarrhythmias. However, whether inducibility of ventricular arrhythmias is predictive of adverse clinical outcome remains unclear.

In summary, to make a diagnosis of SQTS, not only the  length of the QTc, but also the morphology of the T wave and QT adaptation, must be considered, together with personal and family history. The role of electrophysiological study in diagnosis and risk stratification is not yet fully defined.

## Therapy

Despite rapid advances in understanding the genetic basis of SQTS, much less is known about the spectrum of clinical outcomes and the most appropriate treatments for patients with this disease. Investigators have tried a variety of antiarrhythmic agents in an attempt to correct the electrophysiologic abnormalities identified in SQTS patients. Gaita et al [4] gave intravenous flecainide to three SQT1 patients and observed a prolongation of the effective refractory period (from 130-150 ms at baseline to 170-240 ms after flecainide) with suppression of VF inducibility in two of the three patients. Brugada et al [5] gave sotalol to three SQT1 patients and found that QTc remained unchanged. Cellular expression studies showed that the N588K mutation in KCNH2 in these patients reduced binding of sotalol to the I_kr_ channel [5]. Gaita et al [22] then exposed six SQT1 patients to flecainide, ibutilide, sotalol and quinidine. Flecainide caused slight QT prolongation (mainly due to increase in QRS duration); ibutilide and sotalol did not prolong the QT interval. In contrast, quinidine produced normalization of the QT interval, T wave morphology, and ventricular effective refractory period and made VF non-inducible [22]. Wolpert et at later demonstrated that the N588K mutation produced only a 5.8-fold decrease in the I_Kr_-channel blocking effect of quinidine, in contrast to the 20-fold decrease in the effect of sotalol [20]. Other antiarrhythmic drugs that have been tried clinically include propafenone, which suppressed atrial fibrillation but did not lengthen the QT interval [23], and amiodarone, which was used to suppress polymorphic ventricular tachycardia in a SQTS patient with unknown genotype [24]. More recently, disopyramide has been shown to be highly effective in in vitro studies of the N588K-HERG mutation, thus representing a potential alternative for antiarrhythmic treatment [25]. Preliminary data, on two SQT1 patients, carrying  the N588K-HERG mutation, showed that disopyramide increased QT interval, restored the heart rate dependence of the QT interval and increased effective refractory period [26].

The main limitations of all these studies are: 1) the limited number of patients; 2) the homogenous genetic background (only SQT1 patients with the N588K mutation were studied for pharmacological approaches and we do not know if these results are valid also for other SQTS patients); 3) the lack of demonstration of a reduction of spontaneous arrhythmic events with the drugs tested.

In summary, the management of patients with SQTS is still poorly defined. In patients with mutation on the KCNH2 gene, it has been suggested that quinidine(and possibly disopyramide) may be effective in suppressing inducibility at programmed electrical stimulation, but whether this also confers long-term prevention of cardiac arrest is unknown. For the other genetic forms of SQTS, the efficacy of quinidine is less clear.

Up to now, less than 30 cases of SQTS have been reported and the present experience suggests that the disease may be highly lethal. For this reason the implantable cardioverter defibrillator (ICD) is, to date, the therapy of choice in high risk patients with Short QT Syndrome. It should be considered, however, that when only a very limited cohort of patients is available, the severity of a disease tends to be overestimated, as symptomatic patients are identified because of their life-threatening arrhythmias, while asymptomatic patients remain under-diagnosed.

A common complication observed in patients with SQTS treated with an ICD implant is the occurrence of inappropriate shocks, due to tall and narrow T waves characteristic of this disorder [27] and to the prevalence of atrial fibrillation. The management of this disease is further complicated by the fact that sudden death events can occur in the first year of life, as well as in childhood, adolescent age and adulthood and ICD implantation during childhood remains a problem for technical reasons (e.g. problems linked to small body and heart size, difficult vascular access and the need for ongoing modifications of the implant system owing to growth), and for the psychological impact of an ICD in  younger patients [28,29].

Therefore, as no doubts do exist about the indication of an ICD implant in previously symptomatic patients, it may be considered with caution, in young asymptomatic patients, in whom oral quinidine may represent a bridge to ICD implant later in life when technical difficulties are fewer. At present, evidence-based recommendations about management of asymptomatic individuals with a SQTS cannot be made; however, in those with a family history of SCD, inducibility of ventricular fibrillation at the electrophysiological study, may represent a class II indication (level of evidence B) at the ICD implant [30]. In the absence of solid risk stratification criteria, we also suggest that oral quinidine may offer an alternative to an ICD for older asymptomatic SQTS patients, with a negative family history for sudden cardiac death.
